# A Multicenter, Randomized, Double-Blinded, Parallel-Group, Placebo-Controlled Phase I/IIa Study to Evaluate the Efficacy and Safety of a Single Intra-Articular Injection of YYD302 in Patients with Knee Osteoarthritis

**DOI:** 10.3390/jcm11061482

**Published:** 2022-03-08

**Authors:** Yong In, Chul-Won Ha

**Affiliations:** 1 Department of Orthopaedic Surgery, Seoul St. Mary’s Hospital, College of Medicine, The Catholic University of Korea, Seoul 06591, Korea; iy1000@catholic.ac.kr; 2 Department of Orthopaedic Surgery, Stem Cell and Regenerative Medicine Institute, Samsung Medical Center, School of Medicine, Sungkyunkwan University, Seoul 06351, Korea

**Keywords:** osteoarthritis, hyaluronic acid, clinical trial, injection

## Abstract

This study was a phase I/IIa, multicenter, randomized, double-blinded, parallel, placebo-controlled clinical trial that aimed to assess the efficacy and safety of a single intra-articular injection of YYD302, a novel high-molecular-weight hyaluronic acid with divinyl sulfone cross-linking. Thirty adults with knee osteoarthritis were randomized to receive a single 2 mL intra-articular injection of YYD302 (test group 1), 3 mL of YYD302 (test group 2), or 3 mL of the placebo (placebo group). We compared the changes from the baseline in the weight-bearing pain of 100 mm using the Visual Analog Scale (VAS), the Knee Injury and Osteoarthritis Outcome Score (KOOS), the Rheumatology-Osteoarthritis Research Society International (OMERACT-OASRSI) responder rates, and the use of rescue analgesics to assess the safety of this novel drug. A total of 26 subjects (10 in test group 1, 10 in test group 2, and 6 in the placebo group) were included in the full analysis set. At 12 weeks, only test groups 1 and 2 showed significant changes in the weight-bearing pain VAS scores (*p* = 0.0015 and *p* = 0.0085), symptoms, and average daily KOOS values compared to the baseline (*p* < 0.001, *p* = 0.0124, and *p* = 0.0018, *p* = 0.0426, respectively). While the rate and frequency of consuming the rescue drug continued to increase in the placebo group until 12 weeks, there was no change in the test groups. Our findings showed that YYD302, especially 2 mL of YYD302, reduced pain and improved knee joint function compared to the placebo.

## 1. Introduction

Knee osteoarthritis (OA) is a chronic degenerative joint disease characterized by cartilage degradation and bone remodeling [1,2]. OA interferes with daily life due to joint pain and activity limitation, significantly reducing the patient’s quality of life and increasing medical costs [3]. Pain relief is one of the primary goals in the treatment of OA of the knee, and oral medications and intra-articular injections are the known treatment options [1].

Although nonsteroidal anti-inflammatory drugs (NSAIDs) are commonly used for knee OA, they frequently cause gastrointestinal disorders [1]. Steroids have also been used clinically to alleviate knee inflammation, despite the known systemic side effects and the risk of cartilage destruction or infection due to repeated injections [4]. However, these treatment methods can only temporarily relieve pain in patients with OA [1,4].

Hyaluronic acid (HA) is a natural viscoelastic substance with a high molecular weight (HMW) of up to six million Daltons [5]. It is composed of a repeating N-acetylglucosamine and glucuronic acid structure [5]. HA is known to play an essential role in maintaining joint homeostasis and function by producing joint synovial fluid [6]. Viscosupplementation with HA restores the HA concentration and properties of the synovial fluid in the joint cavity and has therapeutic effects, including intra-articular lubricating, anti-inflammatory, analgesic, and chondroprotective effects [7,8,9].

Cross-linked HA has been developed to complement the potency and duration of natural linear HA [10]. Single-injection formulations with increased average molecular weight achieved by cross-linking the HA molecules using various cross-linking agents have been developed to improve patient friendliness and reduce the potential risk of multiple injections. For example, Synvisc-One^®^ (hylan G-F 20, Sanofi-Aventis) is avian-derived HA obtained by the divinyl sulfone (DVS) cross-linking process [11]. In contrast, Synovian^®^ (LG Life Sciences) is an HA derived from bacteria by 1,4-butanediol diglycidyl ether (BDDE) cross-linking [7]. A single injection of cross-linked HA was non-inferior to three weekly injections of HMW HA in terms of weight-bearing pain reduction, suggesting cross-linked HA as an effective and safe treatment for knee OA [7,8,12,13,14,15]. It is known that the increase in viscoelasticity of HA due to cross-linking increases the efficiency of synovial fluid and correlates with the treatment effect in osteoarthritis. Additionally, a single injection of cross-linked HA was superior in terms of cost-effectiveness and improved patient friendliness and potentially reduced the risk associated with multiple injections [16,17].

YYD302 (Yooyoung Pharm Co., Ltd., Seoul, Korea) is a novel single-injection formulation of HA produced by microbial fermentation (*Streptococcus zooepidemicus*) and DVS cross-linking. The purpose of this clinical trial was to assess the efficacy and safety of intra-articular injections of 2 mL and 3 mL of YYD302 before conducting a confirmatory clinical trial.

## 2. Materials and Methods

### 2.1. Subject Population

In this clinical trial, those who met all of the following selection criteria were considered eligible: (1) adults over 40 years old, (2) those who were diagnosed with unilateral or bilateral knee arthritis according to the clinical diagnostic criteria of the American College of Rheumatology (ACR) (if both knee joints of the test subject met the selection criteria, the knee joint with a higher pain VAS score was selected as the target knee. The investigational drug was administered only to the target knee) [18], (3) those who had Kellgren and Lawrence grades [19] of I–III by radiographic examination within 6 months of the screening visit, (4) those with weight-bearing pain VAS scores of more than 40 mm in one or both knee joints, (5) those who had experienced persistent pain despite taking NSAIDs or other pain relievers in the past, (6) those who could walk without a walker or cane (if the patient had been using his/her walking aids routinely for the past 6 months, the patient evaluation could be carried out with the walking aid, under the condition that the walking aid remain unchanged during the study period, and (7) those who voluntarily agreed to participate in the clinical trial.

The exclusion criteria were (1) patients with a body mass index (BMI) of ≥32 kg/m^2^ [20], (2) patients who had been dependent upon psychotropic drugs or narcotic analgesics for more than 3 months, (3) patients who were chronically taking gastrointestinal drugs (for example, H2-blockers, prostaglandin E1, or proton pump inhibitors) and who could not stop taking them during the test period (acetaminophen, the rescue drug in this study, is contraindicated in peptic ulcer patients and may also cause gastrointestinal adverse events), (4) patients with clinically significant abnormal values in the screening blood tests, (5) patients with rheumatoid arthritis or inflammatory metabolic arthritis, (6) patients with serious gastrointestinal disease, liver disease, kidney disease, or heart disease, (7) patients with a joint infection such as septic arthritis, (8) patients with an active skin lesion on the target knee, (9) patients with secondary OA, (10) patients with severe pain such as Sudek’s atrophy, Paget’s disease, or spinal disc herniation that could affect the pain assessment, (11) poly-articular OA patients with severe pain symptoms in other areas (e.g., hip joint,) that could affect the pain assessment of the knee joint, (12) patients who received the following before starting the current trial: HA injection in the target knee within 9 months, HA injection in another joint within 6 months, steroid injections within 3 months, or oral steroids within 1 month, (13) patients with significant joint effusion, (14) patients who underwent arthroscopy for the target knee within the past year, (15) patients who were regularly participating in high-intensity aerobic exercise or heavy-weight anaerobic exercise, (16) patients taking an anticoagulant (except aspirin with a daily dose of 300 mg or less), (17) those with a history of hypersensitivity to investigational drugs, (18) female subjects who were likely to become pregnant during the clinical trial period, (19) pregnant and lactating women, and (20) those who had participated in other clinical trial within 30 days previous to this clinical trial.

### 2.2. Study Design

This clinical trial was designed as a multicenter (two centers), double-blind, randomized, parallel, placebo-controlled, phase I/IIa clinical trial (Trial Registration ID: NCT02965495) and was the first clinical trial to confirm the efficacy and safety of YYD302 in Korea. The study was conducted from 21 November 2016 to 20 September 2017. Subjects who completed the drug washout period for 14 days and met the selection criteria were randomly assigned to test group 1 (test drug 1, 32 mg of sodium hyaluronate gel cross-linked with DVS and 8 mg of sodium hyaluronate fluid; 2 mL of YYD302), test group 2 (test drug 2, 48 mg of sodium hyaluronate gel cross-linked with DVS and 12 mg of sodium hyaluronate fluid; 3 mL of YYD302), or the placebo group (placebo, 3 mL of phosphate-buffered saline) at a ratio of 2:2:1, according to computer-generated randomization using SAS^®^ software version 9.4 (SAS Institute, Cary, NC, USA). The randomization table was designed and generated by a statistician before the clinical trial by sequentially applying permutations of random numbers (A, B, or C) from subject number 1. The participants were administered test drug 1, test drug 2, or placebo once at visit 2.

A designated person, independent of the assessments, administered the investigational drug to ensure double-blindness. The unblinded, designated administrator performed only the administration and recording of investigational drugs for the clinical trial. Safety and efficacy were evaluated at 2 weeks (visit 3), 4 weeks (visit 4), and 12 weeks (visit 5) after administration.

### 2.3. Outcome Measures

The measured outcomes were as follows: changes in weight-bearing pain VAS scores at 4 and 12 weeks after administration compared to the baseline; the percentage of subjects showing improved weight-bearing pain at 12 weeks compared to the baseline; changes in Knee Injury and Osteoarthritis Outcome Score (KOOS) [21] values at 4 and 12 weeks; Rheumatology-Osteoarthritis Research Society International (OMERACT-OARSI) response rates [22] at 12 weeks; changes in resting pain and motion pain (100-mm VAS scores) at 4 and 12 weeks; changes in Patient Global Assessment and Investigator Global Assessment (100-mm VAS) scores at 4 and 12 weeks; changes in knee joint swelling, tenderness and range of motion at each visit; and the rate of taking rescue medication at each visit after administration (number of subjects who took the rescue drug at least once/number of subjects to be analyzed × 100). Improvement in weight-bearing pain was defined as a reduction in weight-bearing pain by at least 20 mm using the pain VAS compared to the baseline, or at least 40% improvement compared to the baseline at 12 weeks.

The data analysis for efficacy was performed using the full analysis set (FAS), and the analysis for safety was performed using the safety set (SS). The FAS group consisted of subjects administered the investigational drug with data available for at least one efficacy endpoint. The safety analysis group consisted of all subjects who received the investigational drug after randomization.

All adverse events (local adverse reactions at the injection site and adverse events outside the injection site) were investigated, regardless of a causal relationship with the investigational drug during the clinical trial period.

### 2.4. Statistical Analysis

For the continuous data, descriptive statistics (number of subjects, average, standard deviation, minimum, median, and maximum) are presented. Frequency and percentage are presented for the categorical variables. The chi-squared or Fisher’s exact test was used to analyze the difference between the groups. The frequency and percentage are presented. If necessary, 95% two-sided confidence intervals were calculated and presented. An alternative Last Observation Carried Forward (LOCF) method was applied. Missing values were not corrected for safety evaluation variables such as physical examination, vital signs, electrocardiogram, or clinical laboratory examination. All statistical analyses were performed using SAS^®^ software version 9.4 (SAS Institute, Cary, NC, USA).

## 3. Results

### 3.1. Subjects Distribution and Baseline Demographics

**Figure 1:**
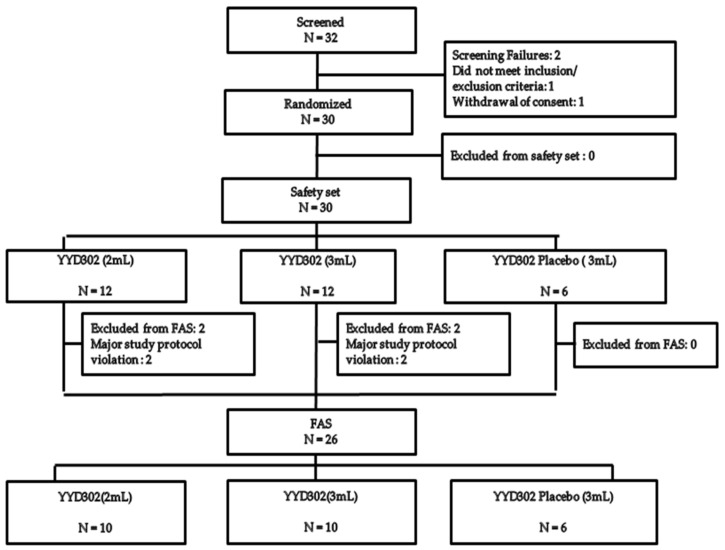
Subject distribution.

Among the 32 subjects who agreed to participate in this trial, a total of 30 patients were finally included in the study (1 subject who failed to meet the inclusion criteria was later excluded, and 1 subject withdrew consent before randomization). All of the 30 subjects were included in the safety analysis, which consisted of 12 subjects in test group 1, 12 subjects in test group 2, and 6 subjects in the placebo group. Of the 30 randomized subjects, excluding 4 subjects with protocol violations (2 subjects in test group 1 and 2 subjects in test group 2), the data of 26 patients were used for FAS analysis. These 26 patients consisted of 10 patients in test group 1, 10 in test group 2, and 6 in the placebo group (Figure 1).

There was no statistically significant difference in the male-to-female ratio among the three groups. The average age of all subjects was 62.60 ± 8.68 years (range, 44 to 80 years). There was no statistically significant difference in the mean age among the three groups. In all three groups, 83.3% of the subjects had OA in both knee joints. There was no statistically significant difference in the mean duration of the disease among the three groups. There was no statistically significant difference in Kellgren and Lawrence stage distribution among the 3 groups (Table 1).

### 3.2. Efficacy Results

The changes in weight-bearing pain at 4 weeks significantly decreased in all groups compared to the baseline (*p* = 0.0016, *p* = 0.0183, and *p* = 0.0287, respectively). Changes in weight-bearing pain at 12 weeks compared to the baseline significantly decreased in the 2 test groups, but not in the placebo group (Table 2).

The changes in the KOOS symptoms and average daily level (ADL) scores showed significant changes in the test groups at 12 weeks, but not in the placebo group (Table 3). Significant changes were found in the test groups for symptoms and average daily level (ADL) subscales. In the pain and sports and recreation (Sport/Rec) categories, test group 1 and test group 2 showed statistically significant changes at 4 weeks post-administration. In comparison, only test group 1 showed statistically significant changes at 12 weeks post-administration (Table 3).

The OMERACT-OASRSI responder rate at week 12 was 70.0% (7/10 patients) in test group 1, 40.0% (4/10 patients) in test group 2, and 50.0% (3/6 patients) in the placebo group, showing no statistically significant difference between each test group and the placebo group (*p* = 0.6066 and *p* = 1.0000, respectively) (Table 4).

A comparison of changes in the Patient Global Assessment (100-mm VAS) scores relative to the baseline between each test group and the placebo group did not show statistically significant differences at week 4 (*p* = 0.7464 and 0.1464, respectively) or week 12 (*p* = 0.9701 and 0.6122, respectively).

**Figure 2:**
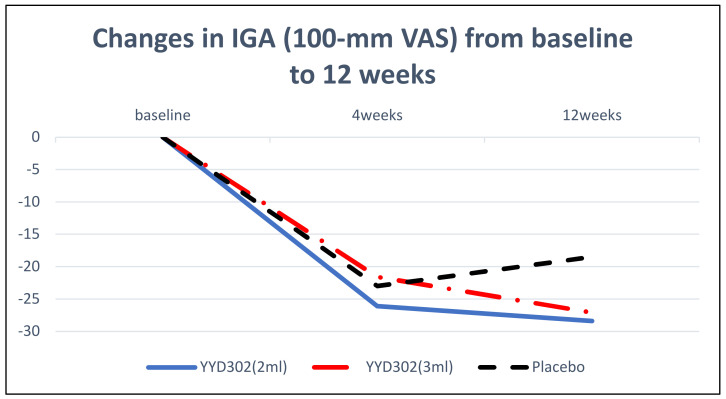
Changes in the Investigator Global Assessment (100-mm VAS) scores from the baseline to 12 weeks.

The changes in the Investigator Global Assessment (100-mm VAS) scores at 4 weeks compared to the baseline were −26.10 ± 21.39, −21.60 ± 16.73, and −23.00 ± 29.26 for test group 1, test group 2, and the placebo group, respectively, showing statistically significant decreases in both test groups (*p* = 0.0039 and *p* = 0.0027, respectively). The changes in the Investigator Global Assessment (100-mm VAS) scores at 12 weeks compared to the baseline were −28.40 ± 24.74, −27.10 ± 18.34, and −8.50 ± 32.46 for test group 1, test group 2, and the placebo group, respectively, showing a significant decreases in both test groups (*p* = 0.0055 and *p* = 0.0012, respectively), but not in the placebo group (Figure 2).

After administering the investigational drug, the rate of using the rescue medication was higher in the placebo group than in the test groups at 4 weeks and 12 weeks. In particular, at 12 weeks, the rate of taking the rescue medication was 40.00% (4/10 patients) in test group 1, 40.00% (4/10 patients) in test group 2, and 83.3% (5/6 patients) in the placebo group, with no statistically significant difference between each test group and the placebo group (*p* = 0.1451 and *p* = 0.1451, respectively).

### 3.3. Safety Results

The total local adverse events and local adverse events at the injection site (pain, warmth, erythema, and swelling), regardless of the causal relationship with the investigational drug during the clinical trial period, are summarized in Table 5. Among the local adverse reactions at the injection site, 7 local adverse reactions were judged as severe, including 3 cases of pain (3/12 patients) in test group 1, 2 cases (2/12 patients) of pain in test group 2, and 1 case (1/6 patients) of pain and 1 case (1/6 patients) of swelling in the placebo group. There were 3 adverse events outside of the injection site, including 2 (2/12) in test group 1 and 1 (1/6) in the placebo group (Table 5). All adverse reactions at the injection site and outside of the injection site recovered uneventfully, except for 1 case of hyperplastic cholecystopathy in test group 1.

In the laboratory tests following the investigational drug administration, no clinically significant change was observed in any group. No subject in the three groups showed a clinically significant change in vital signs, physical examination, or electrocardiogram results after the administration of the investigational drug.

## 4. Discussion

Various HA injections have been considered as relatively safe and useful treatment methods for OA patients not responding to exercise therapy or conservative treatment and who have difficulty taking drugs due to systemic or digestive diseases. Some HA products are extracted from the rooster comb [23], and these can have approximately a 1–3% incidence of hypersensitivity [24].

Recently, to improve patient-friendliness and reduce the potential risk due to multiple invasions compared to existing protocols, a single-injection product line with an increased average molecular weight and viscoelasticity was developed and approved for cross-linked HA using various cross-linking agents [7,23,24,25,26,27]. YYD302 was the first 253 HMW HA extracted by microbial fermentation and formulated by DVS 254 cross-linking for the treatment of human knee OA. DVS has a shorter molecular structure than BDDE; thus, the DVS-HA bonded form creates shorter intermolecular distances than the BDDE-HA related type, resulting in a structurally dense form. The advantage is that it can remain more stable within the joint.

In this randomized, double-blind, placebo-controlled, parallel, multicenter, phase I/IIa trial, the test groups (2 mL of YYD302 and 3 mL of YYD302) showed significantly decreased weight-bearing pain at 12 weeks after administration of the investigational drug when compared to the baseline. The test groups showed better scores on the symptom and ADL subscales of the KOOS, as well as significant Investigator Global Assessment (100-mm VAS) changes at 12 weeks when compared to the placebo group. In particular, considerable pain improvement and knee joint function improvement in the efficacy evaluation (according to the pain and sports and recreation function subscale scores of the KOOS, as well as changes in motion pain) were observed in test group 1 (2 mL of YYD302) compared to test group 2 (3 mL of YYD302).

By default, the 2-mL dosage in test group 1 was set as the minimum efficacious volume by preclinical efficacy results, and the 3-mL dosage in test group 2 was established by the maximum volume of other single injections of HMW HA [7,26]. In the comparison of efficacy results between test groups 1 and 2, test group 1 (2 mL of YYD302) showed more significant changes in weight-bearing pain (100-mm VAS) than test group 2 (3 mL of YYD 302). Changes in the KOOS, resting pain (100-mm VAS), motion pain (100-mm VAS), and the Investigator Global Assessment (100-mm VAS) scores were also more prominent in test group 1 than in test group 2.

Minimal clinically important differences in the VAS scores in knee OA were reported to be between 15 and 20 in previous studies [27,28]. In this study, the changes in VAS scores at 12 weeks in both test groups exceeded this value. However, there was no difference between the test groups. YYD302 is a product designed to have higher physical properties (viscoelasticity) than other single-dose treatments, with a value that is about twice as high. With this characteristic, it is believed that it can stably respond to joint movements and maintain its shape. However, other studies reported that the administration of HA in the articular cavity exceeded 4 mL because of gel swelling, which poses the risk of compression and increasing pain in the articular cavity [29,30]. Therefore, considering intraarticular fluid dosage and fluid swelling, 2 mL is considered a more appropriate dose than 3 mL.

Additionally, the rate of the consumption of the rescue drug gradually increased in the placebo group compared to the test groups. IGA was relatively improved in the YYD302 administration groups compared to the placebo group. However, the magnitude of the clinical differences assessed between the groups was not statistically significant. Therefore, further phase III clinical trials are necessary to confirm the efficacy of the treatment.

Our current study had a relatively small sample size. However, as in the phase I/IIa trial, the sample size was not determined by statistical considerations. We only measured the rate of rescue medications and not the dose in each group. More than 12 weeks of follow-up was not performed, which could be perceived as a weakness of the study. We also did not adopt an active control group that would verify our study drug compared to the placebo.

## 5. Conclusions

In conclusion, YYD302 reduced pain and improved the degree of knee joint function compared to the placebo group. In particular, this positive effect was more prominent in the 2 mL YYD302 group than in the 3 mL YYD302 group. Thus, the 2 mL dosage was more suitable than the 3 mL dose (HA, 40 mg).

## Figures and Tables

**Table 1:** Demographics and clinical characteristics (randomized set).

Characteristics		YYD302 (2 mL) N = 12	YYD302 (3 mL) N = 12	YYD302 Placebo (3 mL) N = 6	Total N = 30
Gender	N	12	12	6	30
	Male, n (%)	1 (8.3)	4 (33.3)	1 (16.7)	6 (20.0)
	Female, n (%)	11 (91.7)	8 (66.7)	5 (83.3)	24 (80.0)
	*p*-value	1.0000 *^,2^	0.6148 ^†,2^		0.3816 ^‡,2^
Age (year)	N	12	12	6	30
	Mean ± SD	60.7± 6.9	65.0 ± 8.3	61.7 ± 12.5	62.6 ± 8.7
	Median	60.5	63.5	59.0	61.5
	Min, Max	44.0, 71.0	52.0, 80.0	44.0, 78.0	44.0, 80.0
	*p*-value	0.8277 *^,1^	0.5064 ^†,1^		0.4693 ^‡,3^
OA site	One-side, n (%)	12	12	6	30
	Left, n (%)	2 (16.7)	1 (8.3)	1 (16.7)	4 (13.3)
	Right, n (%)	0 (0.0)	1 (8.3)	0 (0.0)	1 (3.3)
	Both, n (%)	10 (83.3)	10 (83.3)	5 (83.3)	25 (83.3)
	*p*-value	1.0000 *^,2^	1.0000 ^†,2^		1.0000 ^‡,2^
History of previous administration of nonsteroidal anti-inflammatory agents, OA nutritional supplements, physical therapy, etc.; n (%)	N	12	12	6	30
	Yes, n (%)	0 (0.0)	1 (8.3)	0 (0.0)	1 (3.3)
	No, n (%)	12 (100.0)	11 (91.7)	6 (100.0)	29 (96.7)
	*p*-value	-	1.0000 ^†,2^		1.0000 ^‡,2^
Age at first diagnosis of OA (years)	N	12	12	6	30
	Mean ± SD	58.0 ± 8.5	59.5 ± 5.6	59.7 ± 11.5	58.9 ± 7.9
	Median	57.0	59.0	56.5	58.0
	Min, Max	43.0, 72.0	52.0, 71.0	45.0, 77.0	43.0, 77.0
	*p*-value	0.7306 *^,1^	0.9742 ^†,1^		0.8765 ^‡,3^
Disease duration of OA (years)	N	12	12	6	30
	Mean ± SD	3.3 ± 4.2	6.3 ± 5.7	2.5 ± 2.8	4.3 ± 4.8
	Median	1.5	4.0	1.5	2.0
	Min, Max	0.0, 13.0	0.0, 19.0	0.00, 6.00	0.00, 19.00
	*p*-value	0.7011 *^,1^	0.1501 ^†,1^		0.1859 ^‡,3^
Kellgren and Lawrence I–IV X-ray grading §	N	12	12	6	30
	Stage I, n (%)	2 (16.7)	0 (0.0)	0 (0.0)	2 (6.7)
	Stage II, n (%)	6 (50.0)	6 (50.0)	1 (16.7)	13 (43.3)
	Stage III, n (%)	4 (33.3)	6 (50.0)	5 (83.3)	15 (50.0)
	Stage IV, n (%)	0 (0.0)	0 (0.0)	0 (0.0)	0 (0.0)
	*p*-value	0.2104 *^,2^	0.3156 ^†,2^		0.2193 ^‡,2^

* *p*-value: YYD302 (2 mL) vs. YYD302 placebo (3 mL); ^†^ *p*-value: YYD302 (3 mL) vs. YYD302 placebo (3 mL); ^‡^ *p*-value: YYD302 (2 mL) vs. YYD302 (3 mL) vs. YYD302 placebo (3 mL); *p*-value from ^1^ *t*-test/^2^ Fisher’s exact test; ^3^ ANOVA; § I Based on the target knee in the clinical study; OA = osteoarthritis; NSAIDs = non-steroidal anti-inflammatory drugs; ACR = American College of Rheumatology.

**Table 2:** Changes in the weight-bearing pain (100-mm VAS) at 4 and 12 weeks after administration, compared to the baseline (FAS).

		YYD302 (2 mL) N = 10	YYD302 (3 mL) N = 10	YYD302 Placebo (3 mL) N = 6
Baseline	Mean ± SD	59.8 ± 10.1	56.1 ± 11.1	64.0 ± 12.4
	Median	58.0	56.5	65.0
	Min, Max	44.0, 72.0	40.0, 70.0	46.0, 77.0
W4	Mean ± SD	28.4 ± 18.4	35.6 ± 20.6	29.3 ± 26.1
	Median	28.5	33.0	23.5
	Min, Max	4.0, 60.0	8.0, 77.0	4.0, 70.0
W12	Mean ± SD	25.6 ± 20.8	33.0 ± 21.5	35.0 ± 24.6
	Median	20.5	28.0	31.0
	Min, Max	6.0, 69.0	8.0, 80.0	10.0, 70.0
Change (W4-Baseline)	Mean ± SD	−31.4 ± 22.3	−20.5 ± 22.6	−34.7 ± 27.9
	Median	−35.0	−17.0	−38.0
	Min, Max	−68.0, −3.0	−60.0, 18.0	−72.0, 10.0
	*p*-value (paired *t*-test)	0.0016	0.0183	0.0287
	*p*-value (*t*-test) *	0.7997	0.2834	
Change (W12-Baseline)	Mean ± SD	−34.2 ± 24.1	−23.1 ± 21.8	−29.0 ± 33.2
	Median	−44.5	−22.0	−31.5
	Min, Max	−61.0, 5.0	−62.0, 16.0	−67.0, 10.0
	*p*-value (paired *t*-test)	0.0015	0.0085	0.0850
	*p*-value (*t*-test) *	0.7214	0.6719	

* *p*-value from YYD302 (2 mL) or YYD302 (3 mL) vs. YYD302 placebo (3 mL); SD = standard deviation.

**Table 3:** Changes in the KOOS values at 4 weeks (visit 4) and 12 weeks (visit 5) after administration, compared to baseline (FAS).

			YYD302 (2 mL) N = 10	YYD302 (3 mL) N = 10	YYD302 Placebo (3 mL) N = 6
Total	Change (W4-Baseline)	Mean ± SD	18.3 ± 13.2	9.6 ± 13.1	13.0 ± 18.8
		*p*-value (paired *t*-test)	0.0018	0.0467	0.1528
		*p*-value (*t*-test) *	0.5149	0.6756	
	Change (W12-Baseline)	Mean ± SD	19.8 ± 13.5	11.6 ± 15.2	8.4 ± 22.4
		*p*-value (paired *t*-test)	0.0012	0.0396	0.4014
		*p*-value (*t*-test) *	0.2185	0.7386	
Symptoms	Change (W4-Baseline)	Mean ± SD	21.0 ± 15.5	12.5 ± 14.2	17.3 ± 23.1
		*p*-value (paired *t*-test)	0.0020	0.0214	0.1267
		*p*-value (*t*-test) *	0.6998	0.6143	
	Change (W12-Baseline)	Mean ± SD	25.3 ± 11.7	17.1 ± 17.4	13.1 ± 26.2
		*p*-value (paired *t*-test)	<0.0001	0.0124	0.2753
		*p*-value (*t*-test) *	0.3201	0.7148	
Pain	Change (W4-Baseline)	Mean ± SD	22.8 ± 14.8	13.9 ± 13.8	15.8 ± 20.1
		*p*-value (paired *t*-test)	0.0009	0.0111	0.1126
		*p*-value (*t*-test) *	0.4338	0.8285	
	Change (W12-Baseline)	Mean ± SD	22.0 ± 19.5	9.7 ± 18.1	12.07 ± 27.5
		*p*-value (paired *t*-test)	0.0061	0.1228	0.3320
		*p*-value (*t*-test) *	0.4135	0.8387	
ADL	Change (W4-Baseline)	Mean ± SD	21.3 ± 17.9	14.0 ± 15.04	7.1 ± 25.5
		*p*-value (paired *t*-test)	0.0044	0.0164	0.5252
		*p*-value (*t*-test) *	0.2103	0.5049	
	Change (W12-Baseline)	Mean ± SD	22.9 ± 16.6	13.6 ± 18.2	6.2 ± 28.4
		*p*-value (paired *t*-test)	0.0018	0.0426	0.6190
		*p*-value (*t*-test) *	0.1542	0.5322	
Sports and Recreation function	Change (W4-Baseline)	Mean ± SD	20.0 ± 17.6	10.5 ± 22.7	14.2 ± 21.5
		*p*-value (paired *t*-test)	0.0059	0.1769	0.1682
		*p*-value (*t*-test) *	0.5642	0.7546	
	Change (W12-Baseline)	Mean ± SD	19.0 ± 20.9	12.5 ± 26.5	7.5 ± 28.2
		*p*-value (paired *t*-test)	0.0184	0.1698	0.5441
		*p*-value (*t*-test) *	0.3652	0.7264	
Quality of life	Change (W4-Baseline)	Mean ± SD	6.2 ± 18.2	−3.1 ± 15.7	10.4 ± 11.7
		*p*-value (paired *t*-test)	0.3053	0.5428	0.0797
		*p*-value (*t*-test) *	0.6226	0.0885	
	Change (W12-Baseline)	Mean ± SD	10.0 ± 14.2	5.0 ± 11.7	3.2 ± 12.4
		*p*-value (paired *t*-test)	0.0529	0.2130	0.5614
		*p*-value (*t*-test) *	0.3439	0.7704	

* *p*-value from YYD302 (2 mL) or YYD302 (3 mL) vs. YYD302 placebo (3 mL); KOOS = Knee Injury and Osteoarthritis Outcome Score; SD = standard deviation; ADL = average daily level.

**Table 4:** Changes in the OMERACT-OARSI response rate at 12 weeks after administration, compared to the baseline (FAS).

		YYD302 (2 mL) N = 10	YYD302 (3 mL) N = 10	YYD302 Placebo (3 mL) N = 6
OMERACT-OARSI Response rate	n (%)	7 (70.0)	4 (40.0)	3 (50.0)
	*p*-value *	0.6066	1.0000	

* *p*-value from YYD302 (2 mL) or YYD302 (3 mL) vs. YYD302 placebo (3 mL) by Fisher’s exact test; OMERACT-OARSI = Rheumatology-Osteoarthritis Research Society International.

**Table 5:** Details of adverse events by severity (SS).

			YYD302 (2 mL) N = 12		YYD302 (3 mL) N = 12		YYD302 Placebo (3 mL) N = 6	
System Organ Class *	Preferred Term *	Severity	No. of Patients (%)	Events	No. of Patients (%)	Events	No. of Patients (%)	Events
Overall local AEs		Overall	10 (83.3)	21	9 (75.0)	19	3 (50.0)	9
		Mild	4 (33.3)	5	7 (58.3)	10	2 (33.3)	4
		Moderate	8 (66.7)	13	5 (41.7)	7	3 (50.0)	3
		Severe	3 (25.0)	3	2 (16.7)	2	2 (33.3)	2
Overall local AEsat injection sites		Overall	9 (75.0)	19	9 (75.0)	19	3 (50.0)	8
		Mild	3 (25.0)	4	7 (58.33	10	2 (33.3)	3
		Moderate	8 (66.7)	12	5 (41.7)	7	3 (50.0)	3
		Severe	3 (25.0)	3	2 (16.7)	2	2 (33.3)	2
	Pain	Overall	8 (66.7)	8	9 (75.0)	9	3 (50.0)	3
		Mild	0 (0.0)	0	2 (16.7)	2	0 (0.0)	0
		Moderate	5 (41.7)	5	5 (41.7)	5	2 (33.3)	2
		Severe	3 (25.0)	3	2 (16.7)	2	1 (16.7)	1
	Warmth	Overall	4 (33.3)	4	7 (58.3)	7	2 (33.3)	2
		Mild	1 (8.3)	1	6 (50.0)	6	1 (16.7)	1
		Moderate	3 (25.0)	3	1 (8.3)	1	1 (16.7)	1
		Severe	0 (0.0)	0	0 (0.0)	0	0 (0.0)	0
	Erythema	Overall	2 (16.7)	2	1 (8.3)	1	1 (16.7)	1
		Mild	1 (8.3)	1	1 (8.3)	1	1 (16.7)	1
		Moderate	1 (8.3)	1	0 (0.0)	0	0 (0.0)	0
		Severe	0 (0.0)	0	0 (0.0)	0	0 (0.0)	0
	Swelling	Overall	5 (41.7)	5	2 (16.7)	2	2 (33.3)	2
		Mild	2 (16.7)	2	1 (8.3)	1	1 (16.7)	1
		Moderate	3 (25.0)	3	1 (8.3)	1	0 (0.0)	0
		Severe	0 (0.0)	0	0 (0.0)	0	1 (16.7)	1

* MedDRA (v19.0); AE = adverse event.

## Data Availability

The data presented in this study are available on request from the corresponding author. The data are not publicly available due to privacy and confidentiality.
